# Arthroscopic management of refractory Osgood–Schlatter disease: a case series and surgical technique

**DOI:** 10.1093/jscr/rjaf990

**Published:** 2025-12-17

**Authors:** Abdulelah K Alqawlaq, Mohammad Maddah

**Affiliations:** College of Medicine and Surgery, Batterjee Medical College, Prince Abdullah Al‑Faisal Street, North Obhur, 6231, Jeddah 21442, Saudi Arabia; Musculoskeletal Center, International Medical Center, Hail Street, Al-Ruwais, 2172, Jeddah 21451, Saudi Arabia; Orthopedic Section, Department of Surgery, University of Tabuk, King Faisal Road, Tabuk 47512, Saudi Arabia

**Keywords:** Osgood-Schlatter disease, arthroscopy, ossicle excision, tibial tubercle, athletes, case series

## Abstract

Osgood–Schlatter disease (OSD) is a self-limiting traction apophysitis in adolescents. While most resolve with conservative care, a subset of skeletally mature athletes may develop chronic anterior knee pain from persistent ossicles. Surgical excision is considered when nonoperative measures fail. This study aims to report outcomes of unresolved OSD treated with arthroscopic tibial ossicle excision. Three male patients, aged 34, 45, and 51, presented with anterior knee pain persisting for over one year despite standard non-surgical treatments. Imaging confirmed tibial ossicles. Arthroscopic removal was performed using anteromedial and anterolateral portals under fluoroscopic guidance. All surgeries were uneventful. Patients ambulated immediately postoperatively and returned to sports within 4–5 weeks. At 6-month follow-up, they were pain-free, could kneel comfortably, had full range of motion, and no tibial tubercle tenderness. No complications occurred. Arthroscopic excision is a safe, minimally invasive alternative to open surgery, allowing early recovery and return to sport.

## Introduction

Osgood–Schlatter disease (OSD) is a common cause of anterior knee pain especially during growth spurts. It is mainly due to traction on the tibial tubercle. Most of the time, it resolves with conservative measures including rest, physical therapy, NSAIDs, and reducing physical activities [1].

In some cases, particularly among active, skeletally mature athletes, pain persists. The formed ossicle can interfere with movements and cause discomfort during training, kneeling and pressure which may not always respond to conservative treatment [2].

In such cases, surgery needs to be considered. The classical method is open surgery through the patellar tendon to take out the ossicles and reshape the tibial tuberosity. But in the last few years, arthroscopy has become more common. It is less invasive, leads to smaller scars, and often helps athletes recover faster [3, 4].

From 2018 onward, several papers have described good results with arthroscopic excision [3–6]. These include quick recovery, less downtime, and fewer complications. One mid-term study in 2023 backed this up [3].

## Case report

### Patient and method

We treated three male patients, aged 34, 51, and 45 with unresolved OSD between 2023 and 2025. All had been dealing with ongoing pain just below the kneecap, centered over the tibial tubercle, for more than 6 months even after trying rest, physiotherapy, and other conservative treatments.

**Figure 1:**
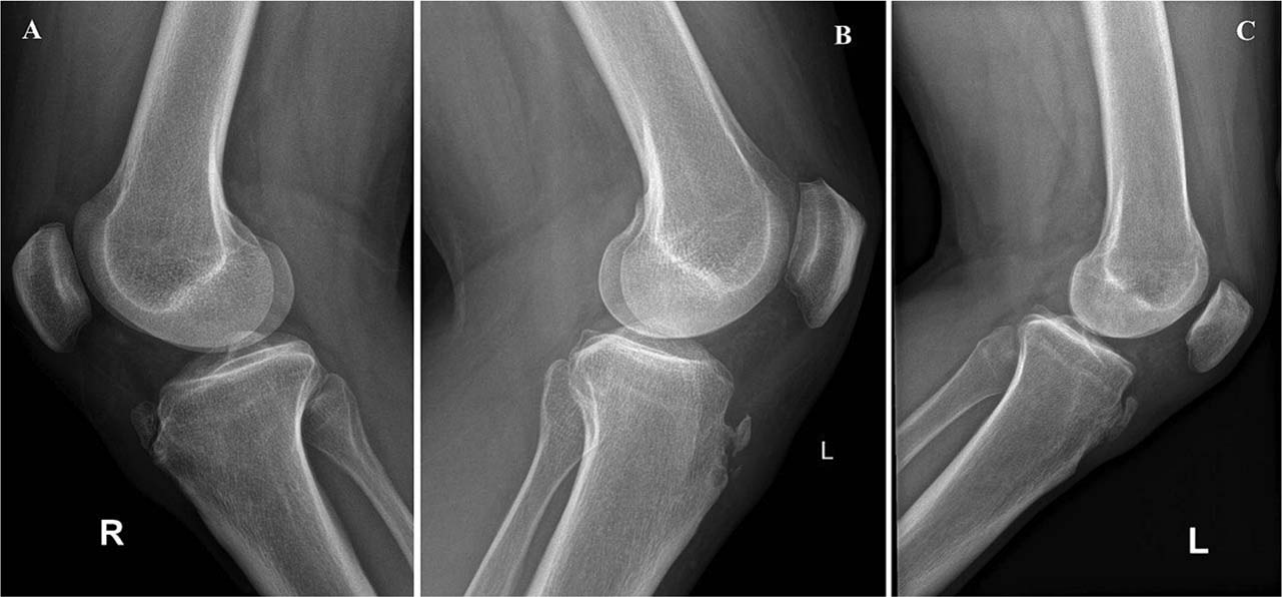
Preoperative lateral radiographs showing persistent tibial tubercle ossicles. (A) Right knee for first case. (B) Left knee for second case. (C) Left knee for third case.

On physical exam, they had localized tenderness at the tibial tubercle, pain when kneeling, and discomfort with resisted knee extension. X-rays showed persistent ossicles in all cases, and we followed up with MRI to rule out any other underlying issues (Fig. 1).

**Figure 2:**
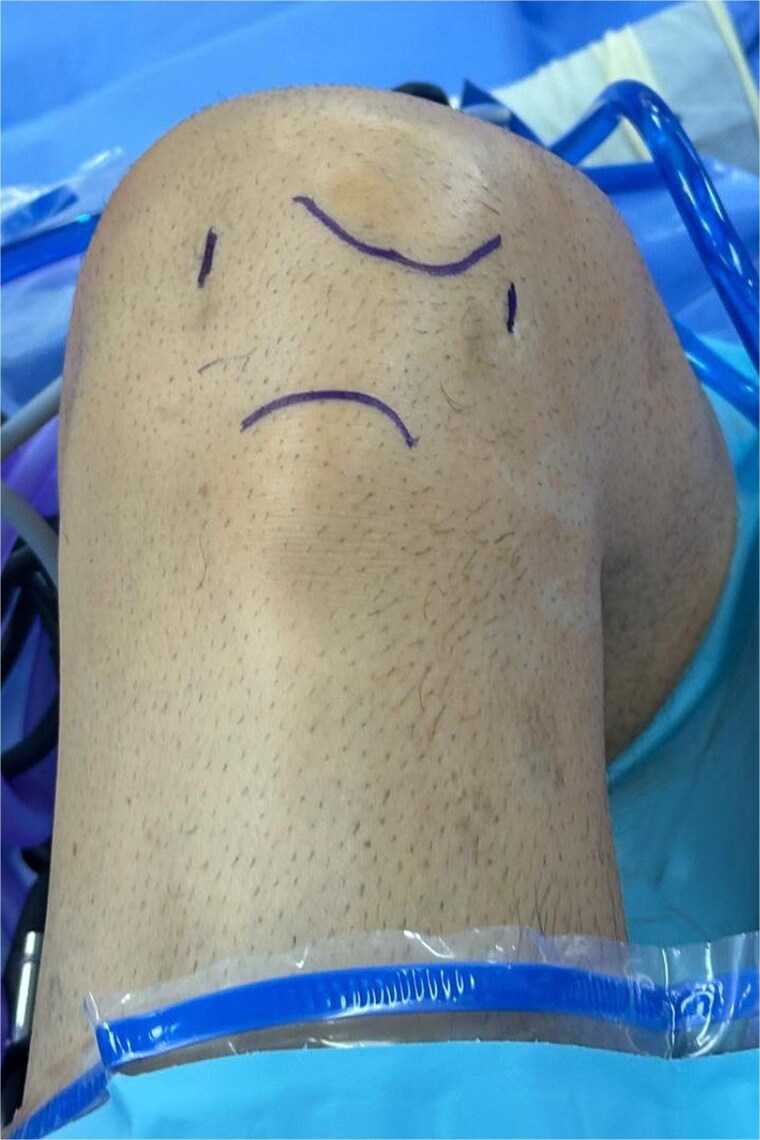
Intraoperative photograph showing arthroscopic portals marked for ossicle excision.

After discussing treatment options, we proceeded with arthroscopic removal of the ossicles. Arthroscopy was performed with the patient in the supine position using superior anterolateral and superior anteromedial portals (Fig. 2).

The procedures were done under either spinal or general anesthesia. Initial diagnostic arthroscopy revealed normal articular cartilage and intact menisci, anterior cruciate ligament (ACL), and posterior cruciate ligament (PCL).

**Figure 3:**
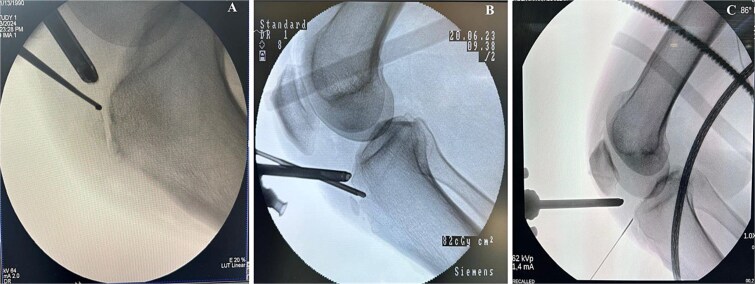
Intraoperative fluoroscopy views show ossicle before excision and complete removal. (A) Case 1. (B) Case 2. (C) Case 3, marking the position of the ossicle using arthroscopic spinal needle.

**Figure 4:**
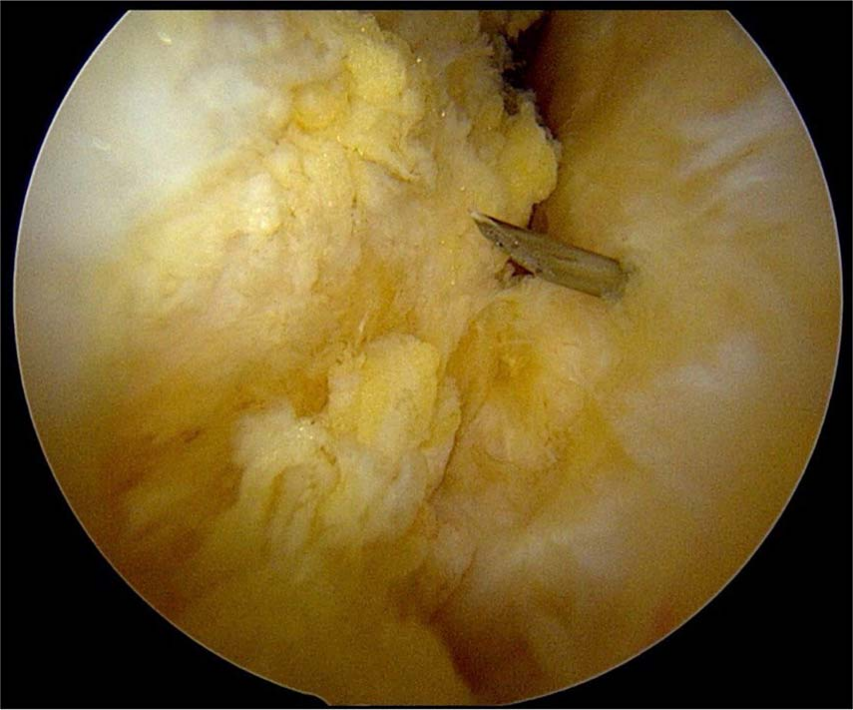
Marking the position with a spinal needle.

Preoperative MRI was used to determine the mediolateral position of the ossicles, using the femoral condyles as landmark relative to the tibial ossicles. Fluoroscopic guidance was also used intraoperatively to identify the level of the tibial ossicle in a lateral X-ray (Fig. 3) with the help of an 18 gauge spinal needle (Fig. 4).

**Figure 5:**
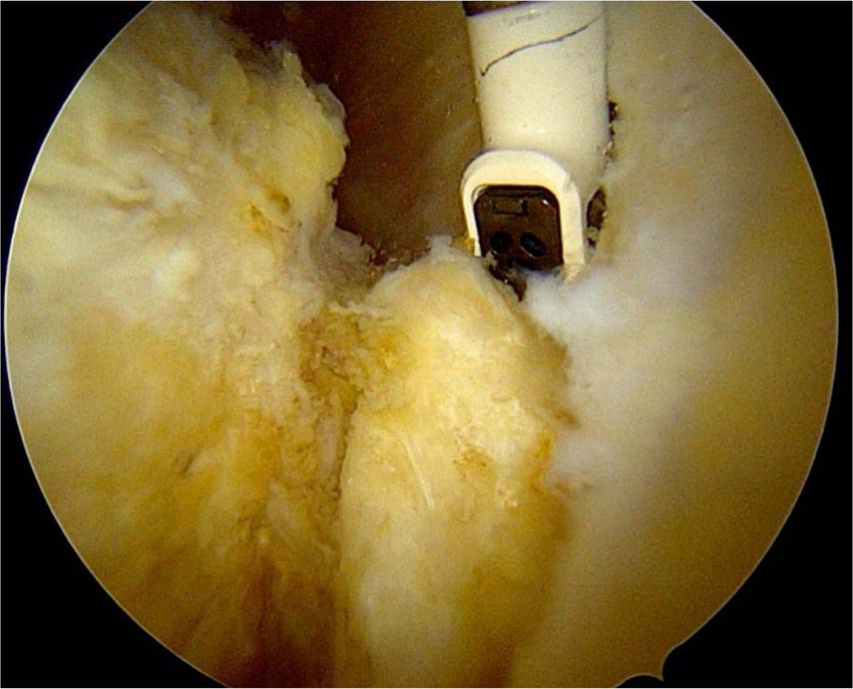
Freeing the ossicle with a radiofrequency probe.

After partial resection of the infrapatellar fat pad for better visualization, the ossicle was exposed by finding the spinal needle and working around it. The fragment was freed arthroscopically using a motorized shaver and a radiofrequency probe (Fig. 5), taking care to protect the patellar tendon.

**Figure 6:**
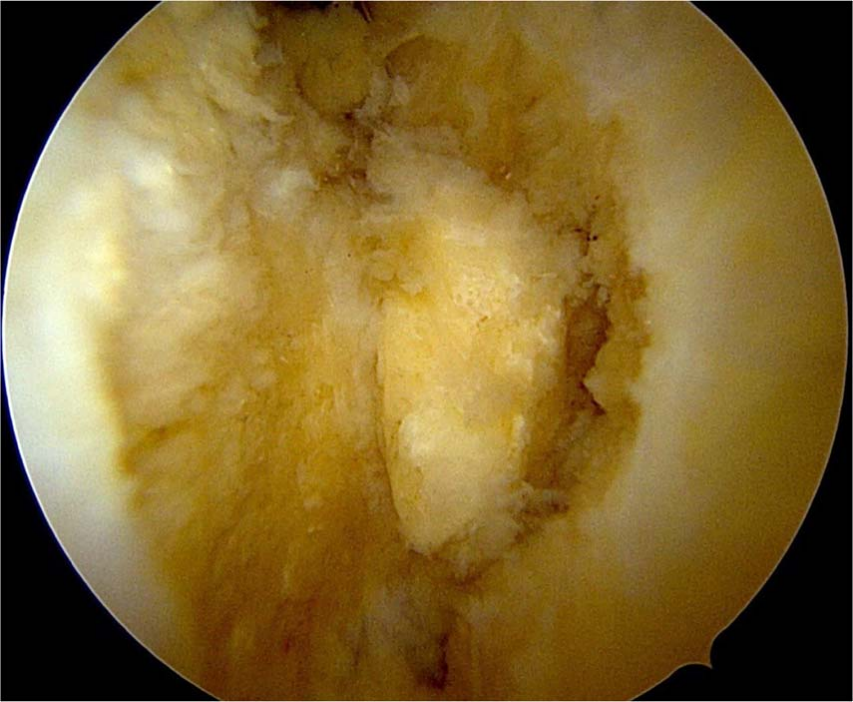
Freeing and mobile ossicle.

**Figure 7:**
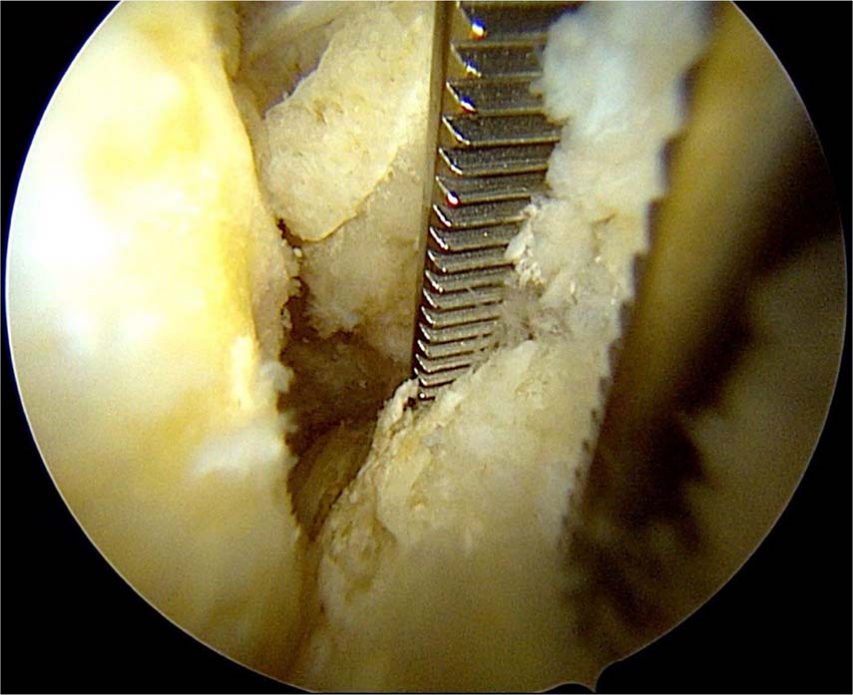
Removal of the ossicle.

**Figure 8:**
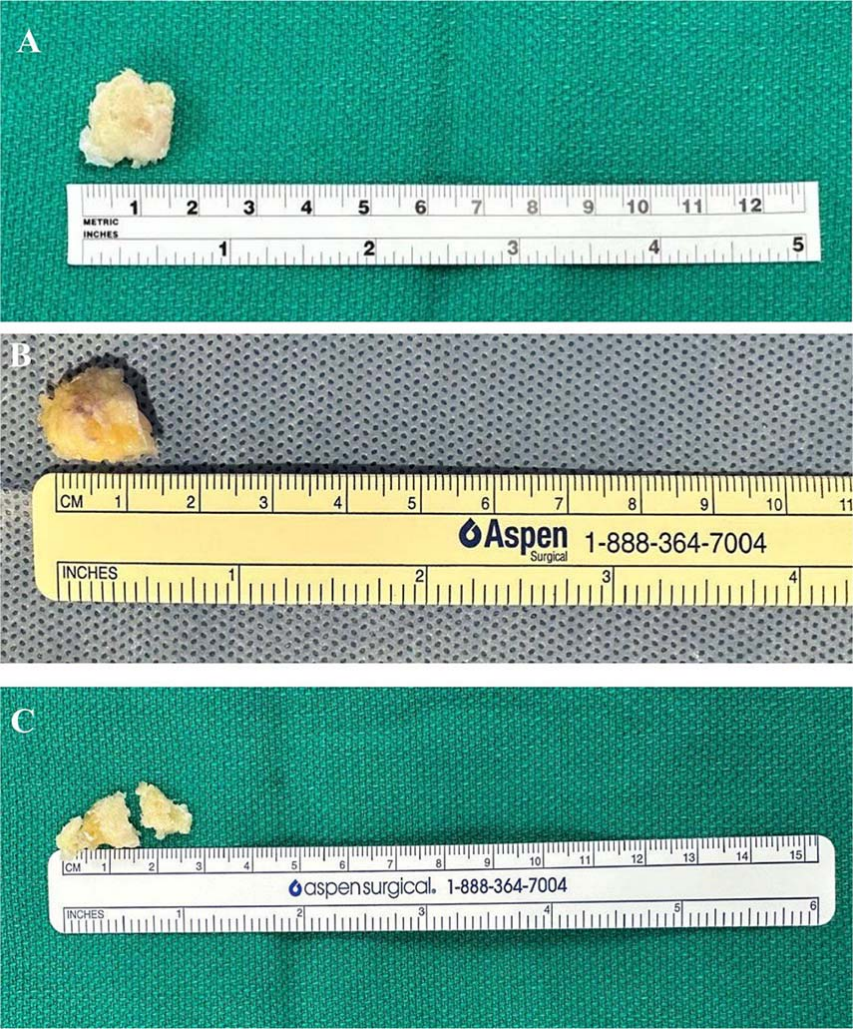
Arthroscopic views of case 1. (A) Ossicle fragment removed. Case 2 (B) tibial tubercle leveling using an arthroscopic Burr. Case 3 (C) ossicle fragment removed.

Once the ossicle was mobile and free (Fig. 6), it was removed either in one piece or was broken into multiple pieces with an arthroscopic burr and then removed with a grasper (Figs 7 and 8). Finally, the tibial tubercle was leveled using either an arthroscopic burr or shaver.

Complete excision was confirmed intraoperatively with fluoroscopy. Hemostasis was achieved by coagulating small synovial vessels, and the procedure was concluded with portal closure.

**Figure 9:**
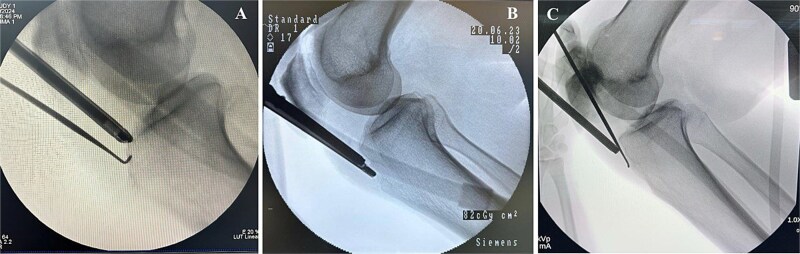
Excised ossicles following arthroscopic removal. (A) Case 1 specimen, measuring approximately 1.5 cm in greatest dimension. (B) Case 2 specimen, measuring ~1.5 cm in greatest dimension. (C) Case 3 specimen broken in two pieces to facilitate removal, measuring ~2.5 cm in greatest dimension.

The excised ossicles were then measured, with dimensions documented as shown in Fig. 9.

Patients were allowed immediate full weight-bearing in knee extension following surgery. Active and passive range-of-motion exercises were initiated on the first postoperative day as tolerated. Quadriceps activation, straight-leg raises, and gentle strengthening began within the first week. Closed-chain strengthening and progressive functional training were introduced between weeks 2 and 4, with return to running and sport-specific drills from weeks 4 to 6, depending on pain and muscle strength. All patients resumed unrestricted activity once swelling had resolved and full quadriceps control was achieved.

This accelerated program aligns with published arthroscopic protocols for unresolved OSD and supports early mobilization with minimal risk of complications [1, 2].

Postoperative recovery was straightforward. All three patients began gentle mobilization and strengthening exercises the day after surgery. One returned to full sports by week 4, and the other two patients by week 6. We followed them for 5–7 months (average: 6 months), focusing on pain levels, kneeling comfort, knee motion, and return to normal athletic routines.

### Results

All three patients tolerated the procedure without complications (Table 1). At final follow-up, all patients reported complete resolution of anterior knee pain and regained full range of motion and strength. None of the patients reported kneeling pain or activity-related discomfort. No intraoperative or postoperative adverse events occurred, corresponding to a 0% complication rate in this series.

**Table 1:** Clinical characteristics and outcomes of the three cases

Case	Age (years)	Side	Symptom duration (months)	Ossicle size (cm)	Kujala Score (pre → post)	Return to sport (weeks)
1	34	Right	12	1.5	83 → 99	4
2	45	Left	8	1.5	81 → 100	5
3	51	Left	10	2.5	85 → 100	6

**Figure 10:**
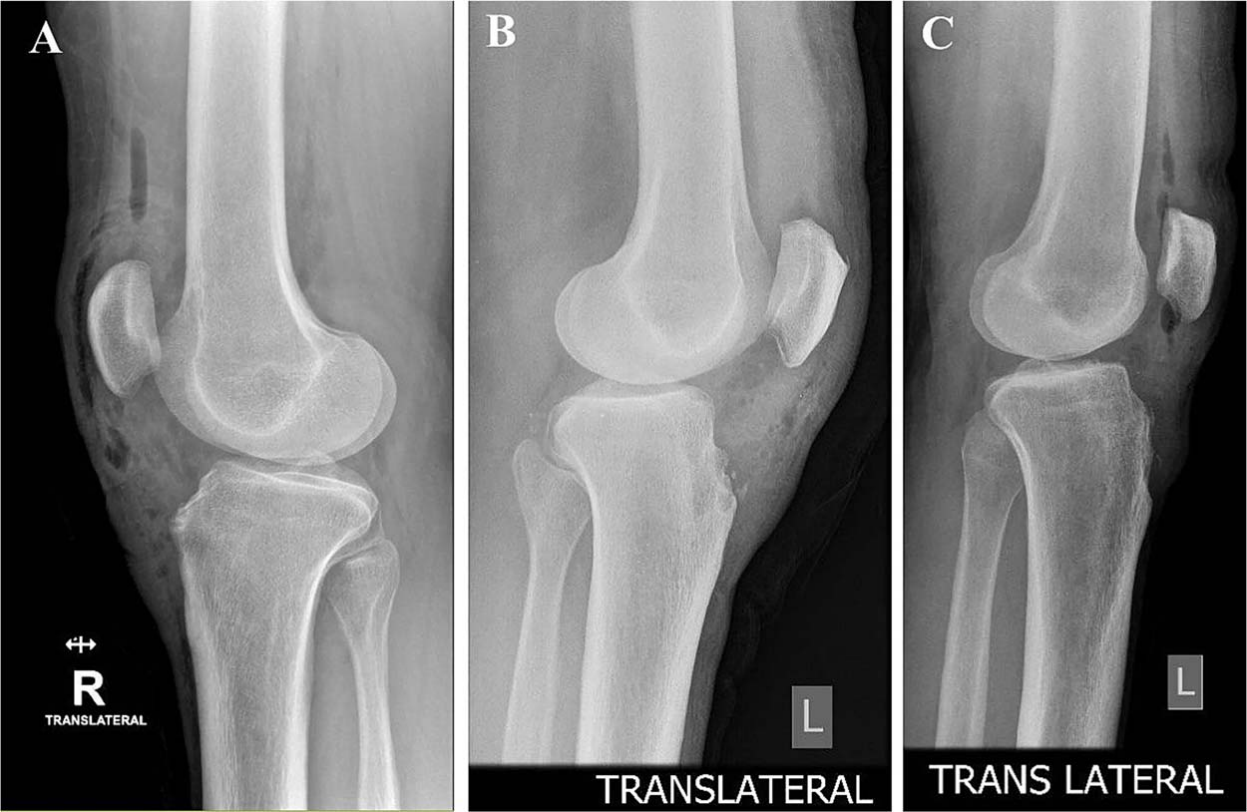
Postoperative lateral radiographs confirming complete excision and smooth tibial tubercle contour. (A) Right knee, case 1. (B) Left knee, case 2. (C) Left knee, case 3.

The mean time to return to unrestricted sports was 5 weeks (range, 4–6 weeks). Radiographs confirmed complete ossicle excision (Fig. 10), and all three patients resumed pre-injury levels. No recurrence or secondary procedures were needed. The Kujala scores improved from 83 to 99, 81 to 100, and 85 to 100, respectively.

## Discussion

This case series reports the results of 3 male patients who had unresolved OSD and underwent arthroscopic ossicle excision. All patients had complete pain relief and got back to sports relatively quickly, without any complications. Our results line up well with previous studies on arthroscopic treatment [7, 8].

Usually, OSD subsides during adolescence. But in some cases, lingering ossicles can keep causing pain well into adulthood, especially in active individuals. When this happens, conservative measures often are not enough, and surgery may be needed [9, 10].

Open excision remains an accepted treatment for unresolved OSD, but it is not without drawbacks. Because the patellar tendon is often split, elevated, or dissected to gain access to the ossicle, tendon repair is usually required, which prolongs recovery and carries a risk of tendon morbidity. Furthermore, open approaches are associated with increased soft-tissue trauma, larger scars, and a higher risk of wound complications, while postoperative immobilization is frequently needed to protect the tendon repair, delaying rehabilitation and return to sport [8]. In contrast, arthroscopic excision avoids direct violation of the patellar tendon, thereby preserving its integrity and reducing the likelihood of tendon-related complications. Care should be taken while working with the shaver around the tendon. We prefer using the radiofrequency ablation while keeping contact to the ossicle to free it from the surrounding soft tissue. If the ossicle is large, elongated, or located deep beneath the patellar tendon, as shown in the third case, it can be broken into smaller pieces using an arthroscopic burr to facilitate its removal.

This minimally invasive approach provides better cosmetic outcomes, minimizes soft-tissue injury, and allows for immediate postoperative mobilization without immobilization, which translates into faster rehabilitation and earlier return to athletic activity. These advantages have been consistently demonstrated in recent series evaluating arthroscopic management of unresolved OSD [7, 10].

Similar success was reported in a group of 11 athletes treated arthroscopically [7], and other small reports appear to echo these results as well [5, 6, 11].

Arthroscopic excision has demonstrated extremely low complication rates, with multiple published series reporting 0% postoperative complications [3, 5–8]. In contrast, open excision where the patellar tendon is typically split or elevated has been associated with higher rates of wound problems (up to 10%–15%) and a greater risk of postoperative stiffness [3, 5–8]. In our series, no intraoperative or postoperative complications occurred, resulting in a 0% complication rate, further supporting the safety of the arthroscopic approach for unresolved OSD.

Overall, our findings support arthroscopic ossicle excision as a safe and effective option for unresolved OSD, providing reliable symptom relief, preserving the patellar tendon, and enabling rapid rehabilitation and return to sport in appropriately selected patients.

## Conclusion

Arthroscopic ossicle excision offers a precise, tissue-sparing solution for unresolved OSD in skeletally mature athletes. Three patients achieved complete symptom resolution, full functional recovery, and early return to sport. These findings support the broader adoption of arthroscopy as a minimally invasive alternative to open surgery in persistent OSD cases.

## References

[1] Ladenhauf HN, Seitlinger G, Green DW. Osgood-Schlatter disease: a 2020 update of a common knee condition in children. Curr Opin Pediatr 2020;32:107–12. 10.1097/MOP.0000000000000842 31714260

[2] Neuhaus C, Appenzeller-Herzog C, Faude O. A systematic review on conservative treatment options for OSGOOD-Schlatter disease. Phys Ther Sport 2021;49:178–87. 10.1016/j.ptsp.2021.03.002 33744766

[3] Akçaalan S, Asiltürk M, Çağlar C, et al. Mid-term outcomes of osgood-schlatter patients undergoing arthroscopic excision. EJM. 2024;63:524–9. 10.19161/etd.1496325

[4] Chandra R, Malik S, Ganti L, et al. Diagnosis and management of Osgood Schlatter disease. Orthop Rev (Pavia) 2024;16:121395. 10.52965/001c.121395 39040500 PMC11262732

[5] Tsakotos G, Flevas DA, Sasalos GG, et al. Osgood-Schlatter lesion removed arthroscopically in an adult patient. Cureus 2020;12:e7362. 10.7759/cureus.7362 32328374 PMC7174857

[6] Kamiya T, Teramoto A, Mori Y, et al. Nano-arthroscopic ultrasound-guided excision of unresolved Osgood-Schlatter disease. Arthrosc Tech 2021;10:e1581–7. 10.1016/j.eats.2021.02.026 34258207 PMC8252821

[7] Circi E, Beyzadeoglu T. Results of arthroscopic treatment in unresolved Osgood-Schlatter disease in athletes. Int Orthop 2017;41:351–6. 10.1007/s00264-016-3374-1 27999926

[8] Mun F, Hennrikus WL. Surgical treatment outcomes of unresolved Osgood-Schlatter disease in adolescent athletes. Case Rep Orthop 2021;2021:6677333. 10.1155/2021/6677333 33815856 PMC7990524

[9] de Lucena GL, dos Santos GC, Guerra RO. Prevalence and associated factors of Osgood-Schlatter syndrome in a population-based sample of Brazilian adolescents. Am J Sports Med 2011;39:415–20. 10.1177/0363546510383835 21076014

[10] Uthraraj NS, Suguru R, Anazor F, et al. Short- to mid-term outcomes in arthroscopic debridement of the knee: a prospective case series. Cureus 2022;14:e32349. 10.7759/cureus.32349 36628030 PMC9826627

[11] Guldhammer C, Rathleff MS, Jensen HP, et al. Long-term prognosis and impact of Osgood-Schlatter disease 4 years after diagnosis: a retrospective study. Orthop J Sports Med 2019;7:2325967119878136. 10.1177/2325967119878136 31700938 PMC6823982

